# Disadvantageous associations: Reversible spatial cueing effects in a discrimination task

**DOI:** 10.1038/srep16156

**Published:** 2015-11-04

**Authors:** Daniele Nico, Elena Daprati

**Affiliations:** 1 Dipartimento di Psicologia, Università degli Studi di Roma “La Sapienza”, Italia; 2 Dipartimento di Medicina dei Sistemi & CBMS, Università degli Studi di Roma Tor Vergata, Italia; 3 IRCCS Fondazione Santa Lucia, Roma, Italia

## Abstract

Current theories describe learning in terms of cognitive or associative mechanisms. To assess whether cognitive mechanisms interact with automaticity of associative processes we devised a shape-discrimination task in which participants received both explicit instructions and implicit information. Instructions further allowed for the inference that a first event would precede the target. Albeit irrelevant to respond, this event acted as response prime and implicit spatial cue (i.e. it predicted target location). To modulate cognitive involvement, in three experiments we manipulated modality and salience of the spatial cue. Results always showed evidence for a priming effect, confirming that the first stimulus was never ignored. More importantly, although participants failed to consciously recognize the association, responses to spatially cued trials became either slower or faster depending on salience of the first event. These findings provide an empirical demonstration that cognitive and associative learning mechanisms functionally co-exist and interact to regulate behaviour.

A good rule to sell a product for a considerable period of time is to create a recognizable pattern for the brand and stick to it. Nowadays, a red can with a white wave shouts the name of a famous soft drink to billions of people, and two golden arches invariably make one think of hamburgers and French fries. Although not necessarily privy to the neural mechanisms involved, the marketing industry has long been aware of the strong associative powers of the human brain. Colour patterns, for example, are known to convey information that automatically refers to a brand’s name, making it readily recognizable^1^,^2^.

In fact, since it was theorized that animals might learn automatically^3^, interest in implicit forms of learning has flourished.

Associative learning is commonly described as a form of learning that takes place whenever a relationship is detected between certain elements in the organism’s environment (such as a cue and an outcome) or between a stimulus and a given behaviour. In this view, previously experienced stimuli readily activate the information with which they have been associated. Computationally, this sort of automatic mechanism is clearly advantageous. For example, in the case of visual perception, stimuli that are deemed to be constant need not be repeatedly processed, thus reducing the brain’s workload. Similarly, making predictions based on previous experience sharpens perceptual abilities: in the case of ambiguous inputs, information gained in previous encounters can provide useful cues^4^. More generally, the human brain is extremely sensitive to regularities in the external world. This ability emerges early in the development, with infants as young as three months old engaging in probability matching to maximize their chances of detecting a target^5^. Besides, the tendency to seek for patterns in external events is so deeply rooted in brain functioning that individuals report having detected regularities in perfectly random sequences^6^ or assume the existence of patterns even when this presumption negatively affects their choices^7^,^8^.

Along these lines, it has been suggested that some “affective tag” is created between information that can be predicted and a positive reward^9^. Namely, individuals seem to actually “prefer” predictable information. In a recent study^10^, participants performed a visual search task in which target location could be/could not be predicted by the position of the presented distractors. Interestingly, when asked about goodness of the displays participants preferred the (easier) predictive ones. More directly, in a controlled study, Trapp and co-workers demonstrated that people attribute a positive value to associative information compared to information that has no or weak associations, even when no apparent benefit emerges^9^. As nicely stated by these authors, “the predictive brain likes things that promote predictions” (p.10). In this view, associative information can be easily described as a precursor of predictive processes in that it minimizes surprise and allows speeding up analysis of incoming inputs.

An interesting aspect of these experiments is that participants are usually unaware of the reasons why they select (or prefer) a given item. In fact, when associative learning is considered, conscious awareness of the underlying link, or rule, is deemed unnecessary and learning is assumed to proceed entirely unaware. For instance, in the serial response time (SRT) task^11^,^12^, participants press a button in response to a visual target that appears in one of four possible locations. Unbeknownst to them, order of target presentation follows a precise (but hidden) rule. By the end of the task, participants are unable to report whether a sequence exists in target presentation. Nevertheless, their response times show that the rule has, in fact, been learnt. A similar pattern is found when regularities involve the temporal association between two visual events: although participants remain unaware of it, different attentional operations are selectively applied according to the detected contingencies^13^, suggesting that conscious perception of regularities in the environment is not necessary for the brain to detect (and apply) them.

Alongside the large body of evidence in favour of forms of implicit learning, a number of cognitive theories have been developed, which mainly describe learning in terms of propositional reasoning and hypothesis testing: namely, by assuming that the link between a cue and an outcome relies on acquisition of a belief about their relationship. A recent review^14^ suggests that awareness is in fact part of the learning mechanism across a variety of conditions, reviving the hypothesis that – in humans – controlled processes play a role also in associative learning^15^. In support of this idea, it is assumed that, accordingly to how it is probed, a certain degree of conscious knowledge exists for most implicitly learnt information (including SRT tasks): several studies indicate that, if tested by free-generation or recognition tasks, implicitly learnt sequences can, in fact, be accessed by participants^16^,^17^.

To account for these contrasting findings, it has recently been hypothesized that a more general distinction could be made between *associative* and *cognitive* processes, wherein the former refers to the construction of links between representations, the latter to the creation and application of rules in a flexible manner^18^. Executive control would here represent the best example of propositional processing. In this dual view, associative and cognitive processes are expected to co-exist, the former mechanism presumably being at work even when more controlled processes are running. This position further suggests that interactions might exist between the two learning modes: specifically, the expression of associative processes might be affected by the degree of involvement of cognitive control exerted on the task^18^.

To directly explore this interaction, we devised a shape-discrimination task, in which participants received *explicit* and *implicit* task-relevant information. The explicit instruction was to press one of two keys in response to a target shape. The target was always preceded by a first event, which – unbeknownst to participants – conveyed *implicit* information (Fig. 1). Structure of the task, and the explicit rule, further provided ambiguous information about the first event that was only *incidentally* defined as a distractor: participants were explicitly told to respond to the second event, thus implying something preceding it. To assess the interaction between cognitive and associative processes, the first event was given the double role of prime and spatial cue: namely, its shape was one of the possible forms of the target, i.e. it was relevant according to the explicit rule (*prime*), while one of its features was implicitly associated to the location where the target would appear (*cue*). The prime, by automatically triggering a response code, ensured engagement of the cognitive system in each trial^19^,^20^,^21^. Besides, as no definite instruction concerning the first stimulus was given, overt search for a rule justifying its role could be expected. In line with the hypothesis that associative processes and cognitive control interact^18^ we predicted a reciprocal modulation of priming and spatial cuing effects. In addition, if interacting implies permeability between the two systems, some information sharing could be anticipated. Accordingly, *detecting* and *using* an implicit association could contribute to the creation of a representation that might lead, at least in some participants, to overt report of the recognized rule.

## Experiment 1

### Results

**Figure 1:**
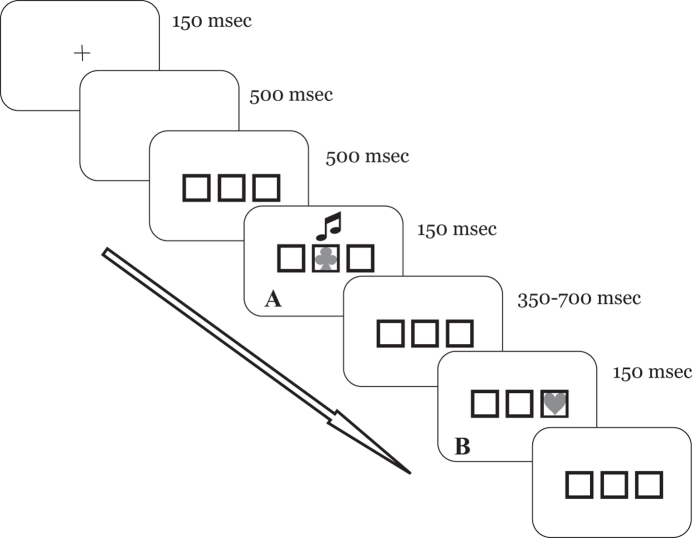
Schematic representation of the trial events in the three experiments. The timeline runs from left to right. Duration of each event is reported on the upper left of each screen representation. Each trial started with a fixation cross, and ended with a view of the empty box triplet. In A is represented the event corresponding to the first stimulus; the musical note depicts either the tone pitch used in Exp.2 or the neutral tone of Exp.3, which was delivered simultaneously with first stimulus’ onset. In Exp.1 no sound was presented and the box including the target (B) was cued through the colour of the first stimulus as in Exp. 3 (see Methods for details).

**Figure 2:**
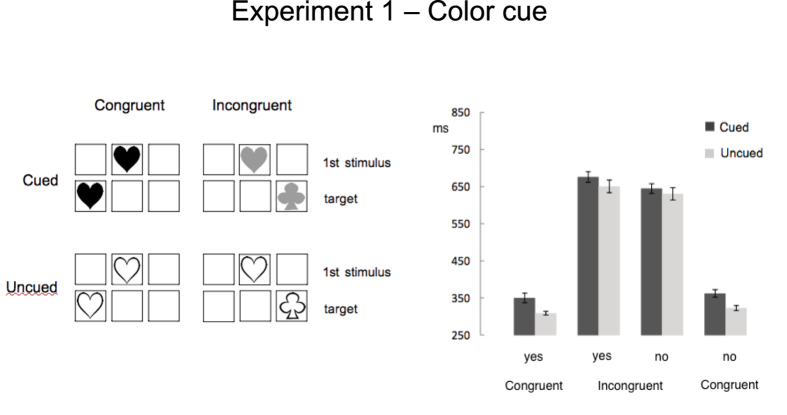
(**a**) In Exp. 1, in cued trials the colour of the first stimulus implicitly suggested target’s location. Congruent/incongruent refers to the correspondence between first stimulus and response. (**b**) Mean correct reaction times and standard errors (in milliseconds) for cued and uncued trials in congruent and incongruent conditions according to response (yes/no). It emerges an effect of priming due to first stimulus’ shape (congruent *vs.* incongruent condition). Spatial cueing produces a robust delay, especially in congruent trials. All conditions differ significantly (*p* < .05).

Sixteen participants (mean age 23.06 ± 1.53yrs) performed the task summarized in Fig. 1. Two successive shapes appeared on a computer screen: the first in the centre, the second in one of two lateral boxes. Participants were explicitly asked to respond on the shape of the second stimulus (“*Is it a club (heart, spade)?*”). Unbeknown to them, colour of the first shape cued the box where the target would appear. There were three colours, two cueing for either lateral box, one favouring none (see Fig. 2a). Manual RTs for correct responses were recorded and analysed.

A summary of results is given in Table 1. ‘Cued’ trials (523 ± 6.7 ms) were significantly slower than ‘uncued’ trials (492 ± 8.9 ms), F(1,15) = 18.96, p = .0006, η_p_^2^ = .56. ‘No’ (504 ± 7.04 ms) and ‘yes’ responses (511 ± 7.24 ms) did not differ, F(1,15) = 4.19, p > .05, η_p_^2^ = .22. RTs in ‘congruent’ trials (i.e. when shape of the first event was identical to that of the target) (342 ± 7.8 ms) were smaller than those in ‘incongruent’ trials (673 ± 15.7 ms), F(1,15) = 261.42, p = .0000001, η_p_^2^ = .95. Two interactions were significant: Congruency X Cueing, F(1,15) = 12.12, p = .003, η_p_^2^ = .45; Congruency X Response, F(1,15) = 27.89, p = .05, η_p_^2^ = .65. Both are summarized in Fig. 2b. The difference between ‘uncued’ and ‘cued’ trials was larger in the ‘congruent’ than in the ‘incongruent’ condition. In addition, in the ‘congruent’ condition, ‘yes’ responses (335 ± 8.61 ms) were faster than ‘no’ responses (349 ± 7.96 ms). The opposite emerged in the ‘incongruent’ condition (‘no’ 659 ± 15.47 ms;‘yes’ 686 ± 16.15 ms;) (see Table 1).

In a debriefing session, none of the participants spontaneously reported to have detected the predictive nature of the first event. When asked about the possible role of the first stimulus, they claimed it had none or justified colour changes as a stratagem to contrast tediousness of trials’ repetition. However, to provide an objective measure of whether participants progressively learned the role of the cue, we explored how its effect varied during the experimental session. The difference (in ms) between cued and uncued trials (Δ_cue_) was compared across blocks by separate t-tests. No differences emerged: a stable, average difference of 32 ± 6 ms was found in each of the 6 blocks (all p > .05).

## Discussion

The first stimulus caused both priming and cuing effects in the absence of overt knowledge that its colour acted as a spatial cue. These findings suggest that both cognitive and associative processes were at play. Although participants did not report active search for a rule, involvement of the cognitive system is supported by the observation that responses to *incongruent* trials (i.e. trials in which the first shape triggered a different response code compared to the target) were significantly slower compared to responses in *congruent* trials (i.e. trials in which first shape and target shared the same response). This is a well-known effect that emerges when a stimulus (S) is paired with a particular response (R). Since the resulting S-R binding is retrieved automatically whenever that stimulus is encountered^22^,^23^,^24^, a certain degree of cognitive control is required to suppress unwanted responses^21^. This results in a correct but slower performance in incongruent trials, as was the case here.

On the other hand, involvement of associative processes is testified by the slowing down of RTs in cued trials. It could be speculated that this delay reflects the interaction between cognitive processes and associative mechanisms, in line with what suggested by previous studies. For example, in an artificial grammar-learning (AGL) task participants receiving explicit instructions about presence of a rule performed more poorly compared to those tested in a fully-implicit learning condition^25^. Similarly, in SRT tasks if individuals receive the general instruction that stimuli are governed by a systematic pattern, their learning is affected, possibly due to the added computational burden derived from intentional search for rules^26^. However, a rule-seeking mechanism evoked by colour should have globally affected RTs, as uncued trials also included a coloured shape. Namely, the cue should have produced a null effect, with comparable RTs in the cued/uncued conditions. Conversely, and quite unexpectedly, the implicit cue produced a selective disadvantage, significantly slowing down RTs only in cued trials. This delay could be due to the interaction between on-going associative and cognitive processes. In fact, the task included a first stimulus to which participants should not respond: independently of whether it shared the shape of the target, participants had to exert control from the beginning of the trial to avoid an early response. In addition, in incongruent trials the effort of suppressing the primed response added to that of controlling the first stimulus. As a consequence, the executive system could not perform the anticipatory orienting of attention to the incoming target suggested by the cue. This would also explain why the delay imposed by the cue was reduced in incongruent trials, i.e. when controlling the response becomes more demanding, making the executive system less prone to the implicit association. We suggest that the observed delay could be considered as the result of a processing that develops serially (namely after that of the shape). By the same token, the stable engagement of cognitive control may have prevented participants to consciously recognize the rule linking colour to target location.

Albeit an explanation suggesting a trade-off between cognitive and associative mechanisms is appealing, the possibility for a more conservative interpretation cannot be ruled out. Here, the first stimulus had two visual features that included relevant information: shape (directly related to the response code) and colour (with its implicit predictive role). As both are conveyed through the visual modality, it is possible that the recorded delay reflects an overload of the visual working memory (WM) system. It has been observed that information that is incidental to the task is maintained in WM and may automatically enter the focus of attention (FOA) if behaviourally relevant^27^. Here, colour of the first stimulus suggests target location: accordingly, one would assume it to be maintained in visual WM. However, due to the larger relevance of shape, information about colour could have been retrieved only *after* target’s appearance. Hence longer RTs in cued trials could reflect a tardive recognition of the association between colour and location (with location prompting colour, rather than the opposite). This in turn would prevent the anticipatory orienting of attention allowing eventually for a confirmatory process^28^. While this mechanism still allows for the association to be created, it deprives the link from any predictive value, possibly explaining why participants remained unaware of the implicit rule. In uncued trials, for which no association exists between colour and target location, responses were faster as no extra-processing was required.

To falsify this hypothesis, in a second experiment we dissociated the sensory modalities through which prime and cue were presented. If the delay reported in cued trials reflects a visual overload, we expected the predictive value of the cue to be restored by using the visual modality for the prime and the acoustic one for the cue.

## Experiment 2

### Results

**Figure 3:**
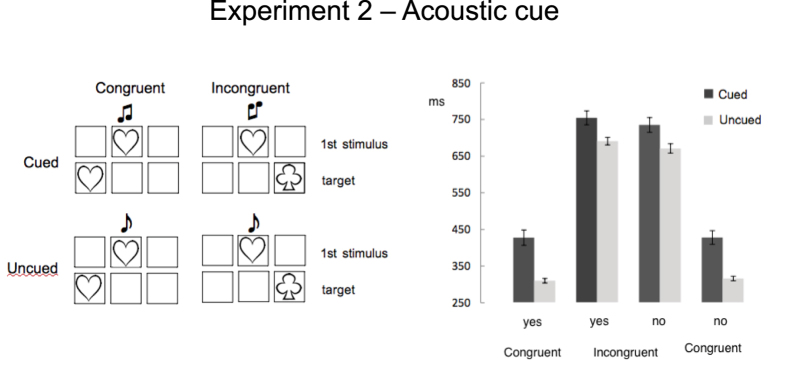
(**a**) In Exp. 2, in cued trials, the pitch of a tone coupled to the first stimulus implicitly suggested the location of the incoming target. As in Exp.1, congruent/incongruent refers to the correspondence between first stimulus and response. (**b**) Mean correct reaction times and standard errors (in milliseconds) for cued and uncued trials in congruent and incongruent conditions according to response (yes/no). An effect of response priming and a severe cue-dependent delay are detected. All conditions differ significantly (*p* < .05) except responses in congruent condition.

Sixteen new participants (mean age 25.88 ± 2.33yrs) were recruited. The task was identical to that of Exp.1 (see Fig. 1) except for the following: the first stimulus was monochromatic and appeared simultaneously with a tone whose pitch coded for target location in a similar way to colour in Exp.1. Namely, there were three different tones: two coded for one of the two lateral boxes, respectively; one allowed no prediction (see Fig. 3a).

As in Exp.1 responses were slower to ‘cued’ trials (602 ± 20.14 ms) than to ‘uncued’ trials (508 ± 6.76 ms), F(1,15) = 35.93, p = .0002, η_p_^2^ = .71. ‘No’ responses were faster (551 ± 12.8 ms) than ‘yes’ responses (560 ± 12.7 ms), F(1,15) = 10.01, p = .006, η_p_^2^ = .40; and RTs to ‘congruent’ trials (375 ± 11.35 ms) were shorter than those to ‘incongruent’ trials (736 ± 15.41 ms), F(1,15) = 1173.37, p = .0000001, η_p_^2^ = .99. Only the interaction Cue X Congruency was significant, F(1,15) = 33.60, p = .00003 η_p_^2^ = .69 (Fig. 3b, Table1). The difference between ‘cued’ and ‘uncued’ trials was larger in the congruent than in the incongruent condition. Although results largely replicated those of Exp.1, in Exp.2 RTs were overall slower, F(1,30) = 9.18, p = .005, η_p_^2^ = .24. Specifically, here RTs to cued trials (but not to uncued ones) were significantly longer (Experiment x Cue, F(1,30) = 10.96, p = .002, η_p_^2^ = .27).

When debriefed, none of the participants reported the predictive nature of the first event. A stable, average Δ_cue_ of 94 ± 8 ms was found in the 6 blocks. Separate t-tests confirmed that this value did not vary across blocks.

## Discussion

Results of the second experiment replicated those of Exp.1. As in the previous case, the first shape induced priming effects. Also, participants remained unaware of the association linking the tone pitch associated to the first stimulus to target location. Crucially, a significant lengthening of RTs was again recorded in cued trials. The latter effect emerged even if prime and cue were conveyed through different sensory modalities, ruling out the possibility that the delay induced by the spatial cue reflects some form of overload.

It appears more likely that the inability to benefit from cuing relies on the interaction between cognitive and associative mechanisms. However, compared to previous studies, which showed a non-specific disruption of associative mechanisms when propositional processes were at play^25^,^26^, here the task was not uniformly affected, as shown by the fact that uncued trials were not slowed down. To account for the present findings we hypothesize that the link between pitch and target location was *detected* but could not be used. A possible reason could be that the implicit spatial association is learnt *across* trials, while the link between first event and target (which includes the strong effect of the prime) is evident from the very beginning of the task, i.e. at a much earlier stage. The type of tasks applied here implies the repetitive execution of a sequence of operations. This creates a functional routine, also described as *task-set*^29^ that can be viewed as a cognitive module incorporating the perceptual and motor processes necessary to perform the task. Automatizing a sequence of operations has the ecological advantage of reducing involvement of the cognitive system, whose intervention can be limited to supervision of routines’ unfolding, task switching or response inhibition. Here, it is reasonable to assume that the operating routine is defined by a sequence including a first event, irrelevant to the response, followed by the target, whose shape is coupled with a response code. Only after several trials, the existence of an association between one characteristic of the first event and target location emerges. Namely, the existence of a regularity becomes available only once the response routine has been firmly established and is currently running. Although routines can be decomposed in functional subsets^30^, updating task-set information demands activation of control processes^31^,^32^,^33^ and reconfiguration of a routine may require re-activation of the running module^33^,^34^. In this view, the selective delay generated by spatial cuing could be ascribed to the impossibility of incorporating this newly acquired piece of information in the main routine. In this sense, the invariance of the delay across blocks (in both Exp.1 and 2) would be in line with the idea of the relative rigidity of the set of operations involved. Moreover, regularities, such as the association between first event and target location, have strong predictive capabilities and represent by themselves salient information^9^,^35^, unlikely to be easily ignored. Similarly to Exp.1, we assume that the delay in responding to cued targets could then reflect the time required to serially process this information. In this view, it could be hypothesized that if information conveyed by the spatial cue were made available at an earlier stage, its predictive value would be restored allowing for target’s anticipation. To test this hypothesis, we replicated the first experiment coupling a neutral tone to the first event in order to increase its saliency through multisensory integration processes^36^.

## Experiment 3

### Results

**Figure 4:**
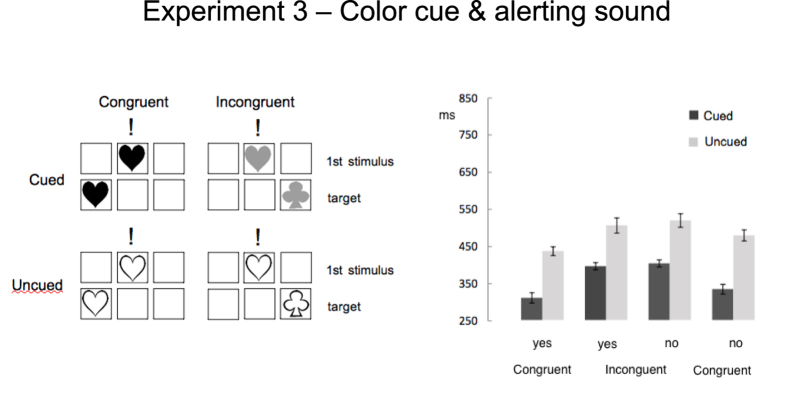
(**a**) In Exp. 3, in all trials the first stimulus was always coupled to a neutral tone (a beep). As in Exp.1, first stimulus’ colour implicitly cued for target’s location. (**b**) Mean correct reaction times and standard errors (in milliseconds) for cued and uncued trials in congruent and incongruent conditions according to response (yes/no). Here spatial cueing allows for a successful anticipation of the target and RTs in cued trials are significantly faster. Only a moderate effect of response priming is present. All conditions differ significantly (*p* < .05) except responses in the incongruent condition.

A third group of sixteen participants (22.63 ± 1.63yrs) was involved. The task was identical to that of Exp.1 (i.e. colour of the first shape implicitly cued for target location, see Fig. 1) but here the first shape was paired to a neutral tone (see Fig 4a).

As in the previous two experiments, the main effect of cueing was significant (Fig. 4b), F(1,15) = 85.21, p = .00001, η_p_^2^  = .85. However, here responses to ‘cued’ trials (366 ± 10–48 ms) were faster than those to ‘uncued’ trials (496 ± 14.26 ms). ‘Yes’ responses (420 ± 10.73 ms) were faster than ‘no’ responses (442 ± 10.81 ms), F(1,15) = 14.15, p = .001, η_p_^2^ = .48. RTs were faster in ‘congruent’ (396 ± 11.67 ms) than in ‘incongruent’ condition (466 ± 13.51 ms), F(1,15) = 22.92, p = .0005, η_p_^2^ = .60. Two interactions were significant: Cueing X Congruency F(1,15) = 8.45, p = .01, η_p_^2^ = .36; Congruency X Response, F(1,15) = 30.31, p < .003, η_p_^2^ = .67. The difference between ‘cued’ and ‘uncued’ trials was larger in the congruent than in the incongruent condition. ‘Yes’ responses were faster than ‘no’ responses only in congruent trials (see Table 1). In addition to the reversed effect of the cue, compared to Exp.1, here RTs were globally faster (F(1,30) = 34.13, p = 000002, η_p_^2^ = .54). This was specifically due to RTs to cued trials, Experiment x Cue interaction, F(1,29) = 97.53, p = 0000001, η_p_^2^ = .76. The effect of the prime also differed, RTs to incongruent trials becoming faster in Exp.3 (Experiment x Congruency, F(1,29) = 101,58, p = 0000001, η_p_^2^ = .77).

None of the participants was aware of the predictive nature of the first event. However, here Δ_cue_ increased from −11 ms in block1 to −113 ms in block2 and −187 ms in block6. Separate t-tests showed significant differences between block1 and 2 (p < .0006) and block4 and 5 (p < .0005).

### Discussion

The main finding of Exp.3 is that the pattern of results produced by the spatial cue was completely reversed since spatial cuing produced a significant advantage. This finding indicates the implicit information linking colour to target location was not only detected, but also proficiently *used*. Besides, shape priming was markedly reduced.

The tone being the only difference compared to Exp.1, we assume that its presence affected the whole organization of cognitive operations involved in the task. It has been shown that in visual search tasks, non-spatial auditory signals favour processing of visual features by affecting selection priority, a phenomenon which is assumed to occur relatively early in the perceptual process^36^. In a similar way here we assume that the addition of the neutral tone allowed all perceptual attributes of the first event to be immediately included in the task operational routine. Furthermore, recent results show that bimodal attentional templates can be created that include *both* visual and auditory features: so, selective attentional processing would not be guided exclusively by visual target-defining features^37^. Accordingly, compared to Exp.1, here factors independent from the intrinsic features of the prime (e.g. the tone) were likely drawn in. Cognitive control was thus exerted earlier against a multimodal distractor rather than being elicited by a shape that may be relevant to the response. This possibility is supported by the observation that – compared to Exp.1 – the effect of the prime was significantly reduced (see Fig. 4b).

The different, less effortful, involvement of cognitive control we hypothesize had a number of important consequences: orienting processes could anticipate target location; responses were faster in all conditions, suggesting a more efficiently paced sequence of operations; a practice effect was observed, with cuing speeding up responses not only from block1 to block2, but up to block5.

## General Discussion

In three experiments, we explored the behavioural effects of providing participants with both explicit instructions and implicit information about the task. To assess whether recruitment of cognitive processes interacts with automaticity of associative mechanisms, we included the incidental information that the target would be preceded by another stimulus. The latter played the double role of prime (i.e. its shape was relevant to the response processing) and (hidden) spatial cue. Participants never explicitly detected that the first event acted as a cue to target location, even if the ambiguous instruction could have favoured intentional rule-seeking^26^. Nevertheless, in all experiments the cue selectively altered responses, either by lengthening (in Exp.1 and 2) or speeding up RTs to cued trials (in Exp.3). This implies that embedded information – if relevant to the task – never went amiss, albeit its exploitation varied across experiments. Specifically, even if associative processes can work in the background, i.e. independently from cognitive ones, the opportunity to benefit from implicitly learnt information was influenced by when and how cognitive control was engaged.

The fact that an *effect of the cue* was always found would be in agreement with the ecological advantage offered by detecting regularities and/or causal relationships among events. Information that can be predicted is processed faster and reduces the computational burden of the brain, allowing for more cognitive resources to be devoted to processing novel information and, eventually, to creating new associations. Indeed, it has been shown that individuals search for patterns even when this behaviour is entirely useless, either because there are no regularities or, if present, they bring no advantage or even interfere with performance^38^,^39^,^40^. With respect to the latter, studies on AGL and SRT tasks^25^,^26^ have shown that providing participants with the information that a rule exists in a seemingly random pattern affects associative learning. In the present study, a lengthening of RTs to cued trials was observed only in Exp.1 and 2, suggesting that an explanation in terms of intentional rule seeking would not entirely account for the participants’ inability to take advantage of the cue. This specificity, together with the relative independence of the interference from the sensory modality involved (color in Exp.1, tone pitch in Exp.2) rather seems to describe a more complex form of interaction.

We suggest that the *differential response to cuing* depends on when and how cognitive control is engaged by the task. In all experiments, the implicit spatial association is learnt across trials. Conversely, the link between first event and target shape is available at a much earlier stage (tapping directly from the explicit instruction) and – by acting on the response – it carries meaningful information. In *Exp.1 and 2*, this organisation may have selectively delayed cued trials. Indeed, the need to control for the effects of the prime is likely to have induced a strong engagement of cognitive processes. As a consequence, when the association between colour/tone pitch and target location was established, the demanding process of incorporating this information in the task-set^29^ could not be performed. The paradoxical lengthening of RTs would thus reflect the time required to serially process the however useful implicit information carried by the cue. Alternatively, it could be speculated that the association was made between target location and cue, rather than the opposite, depriving the latter of its predictive role. In this view it is unsurprising that the regularity failed to reach consciousness: both accounts imply that attention could not anticipate target location. However, it remains that in Exp.1 and 2 the meaningful role of the cue was somewhat detected: to our knowledge, this is the first report showing a selective delay generated by a 100% valid implicit spatial cuing. In *Exp.3*, by increasing saliency of the first event, i) all features of the first stimulus became simultaneously relevant (i.e. participants likely viewed a “red club” rather than “club”+“red”) and/or ii) cognitive control was differently engaged, as shown by the fact that orienting processes took place, i.e. in this condition the spatial cue was both detected and used. However, as in the previous experiments, participants remained unaware of the implicit mechanism. We can speculate that this lack of awareness could depend on the fact that the cue predicted location and was thus only indirectly linked to the response in all conditions.

Taken together, results from the three experiments support the idea that cognitive control and associative learning mechanisms functionally co-exist and interact. As suggested by previous studies^18^,^41^, associative processes were active “in the background” all through the experiments. Depending on when and how cognitive control intervenes, associative mechanisms allowed only for detection of the association or for its detection and use to anticipate the next event. This functional dissociation suggests that the ability to recognize regularities in the environment does not rely on the same mechanisms involved in applying this implicit knowledge. Albeit neuroanatomical speculations are beyond the scope of the present work, this finding is in line with studies showing that distinct neural networks are involved when using already implemented stimulus-response associations or simply instructed ones^42^, and also in representing newly instructed information about stimulus-response mappings as opposed to their actual utilization^43^.

## Methods

### Participants

Three groups of 16 healthy individuals (8 women) participated to the study (mean age, Exp.1: 23.06 ± 1.53yrs; Exp. 2: 25.88 ± 2.33yrs; Exp. 3: 22.63 ± 1.63yrs). All were naive as to the purpose of the experiments, had normal or corrected-to-normal vision and did not suffer from deafness or colour-blindness. Before entering the experimental session all participants signed informed consent. The study was conducted in accordance with the declaration of Helsinki and was approved by the ethical committee of the Department of Psychology of the University of Rome “La Sapienza”.

## Experiment 1

### Stimuli

Participants rested their head on a chin-rest in front of a 24” BenQ VA LED monitor (1920×1080, Tr 8 ms, refresh rate 60Hz) located at a 50-cm viewing distance. A central fixation marker “+” (.41° by .41° wide) was presented at the centre of the screen (Fig. 1). The baseline-display consisted of three boxes (3° wide, 2° eccentricity from each other). First stimulus and target were coloured shapes (1.3° wide) depicting a heart, spade or club appearing in one of the boxes. The red/green/blue (RGB) values for the colours were 0,0,255 (blue); 0,255,0 (green); 255,0,0 (red). Stimuli appeared on a grey background (RGB, 180,180,180).

### Procedure

Participants had to maintain fixation and respond to target shape by pressing as fast as possible one of two vertically aligned keys (response/key correspondence was balanced between subjects). The question “Is the target a club (heart/spade)”? was posed before each block of trials. Instructions explicitly stated that the target was the second stimulus. All trials had the same structure (see Fig. 1), starting with the central cross (150 ms), followed by a 500 ms blank, and three boxes. After 500 ms the first stimulus appeared in the central box for 150 ms. Following an 350–700 ms interval, the target appeared for 150 ms in one of the two lateral boxes. All boxes disappeared after participant’s response or 1500 ms. Inter-trial interval varied between 450–550 ms. All intervals were randomly chosen from a discrete number of values. Participants were asked to avoid eye movements after disappearance of the cross. A JVC-TK240 camera mounted on the computer screen allowed one experimenter to verify that they complied with this instruction.

Colour of the first stimulus cued the box where the target would appear. There were three colours, two cueing for either lateral box, one favouring none (i.e., target appearing with 50% probability in either lateral box; e.g., blue = left box, red = right box, green = right or left box) (see Fig. 2a). Target and first stimulus always shared the same colour.

1152 trials were presented (in 6 separated blocks of 192 trials each): half of them included a spatially informative cue. Expected response was ‘yes’ in half of the trials. Target shape (heart/club) and colour by side coupling were balanced between subjects. Order of presentation of trial types, cued side, and stimulus-target congruency according to response was randomized in each sequence and across participants. Each block lasted approx. 10min. Participants were free to take a brief pause at the end of each block. The whole experiment lasted about 60–75 min. Before starting the experiment, a training session of 10 trials was run to familiarize participants with the task.

A short interview (*debriefing*) was run at the end of the experiment in which participants were asked whether they had any special remark to make about the task (e.g. relating to trials difference in difficulty, stimuli’s features, response accuracy/speed).

## Experiment 2

*Stimuli and procedure* were identical to those of Exp.1 except for the following: the first stimulus was monochromatic and appeared simultaneously with a tone whose pitch coded for target location in a similar way to colour in Exp.1: high/medium/low pitch coding for right, left, or both lateral boxes with equal probability, respectively (see Fig. 3a). Tones were delivered through earphones and were 150 ms square-waves synchronized with first stimulus’ onset. The low/middle/high-pitched tones were of 362/547/732 Hz, respectively.

## Experiment 3

*Stimuli and procedure* were identical to those of Exp.1 except for the following: the first stimulus was paired to a neutral tone. The tone was delivered through earphones and was a 150 ms square-wave synchronized with first stimulus’ onset. A single 500-Hz tone was used.

### Data Analysis

Mean Reaction Times (RTs) for correct responses for each condition were computed. Less than 2% of trials were discarded due to anticipation (response faster than 100 ms), misses (no key-press or response slower than 1500 ms), errors or eye movements. Means and standard errors (SE) are provided in the Results section. For each experiment, RTs were submitted to a 2×2×2 repeated measures ANOVA with cueing (cued/uncued), congruency between first stimulus and target according to response (congruent/incongruent) and response (yes/no) as within-subject factors. Separate t-tests (one-tailed) were run to compare differences between cued/uncued trials and yes/no responses in the different conditions. In addition, two separate ANOVAs with Experiment as between subject factor and Cue, Congruency and Response as within subject factors were ran to compare Exp.1 vs. Exp.2 and Exp.1 vs. Exp.3, respectively. For the sake of brevity, only results related to the main topic of the study (i.e. implicit cuing and priming effects) are reported in the text. Finally, to assess whether and how the observed effects varied across blocks we computed the difference (in  ms) between cued and uncued trials (Δ_cue_) and run separate t-tests across blocks for each experiment.

Alpha level was set at .05 for all tests. Bonferroni corrections for multiple comparisons were applied when appropriate. Prior to running the ANOVAs, normal distribution of the responses was assessed for each group of participants via Shapiro-Wilk W test. For Exp.2 in which data distribution was not normal (W = .83, p = .007), RTs were submitted to logarithmic transformation prior to being entered in the ANOVA. Comparison between results of Exp.1 and 2 was also performed on log transformed data.

## Additional Information

**How to cite this article**: Nico, D. and Daprati, E. Disadvantageous associations: Reversible spatial cueing effects in a discrimination task. *Sci. Rep.* **5**, 16156; doi: 10.1038/srep16156 (2015).

## Figures and Tables

**Table 1:**
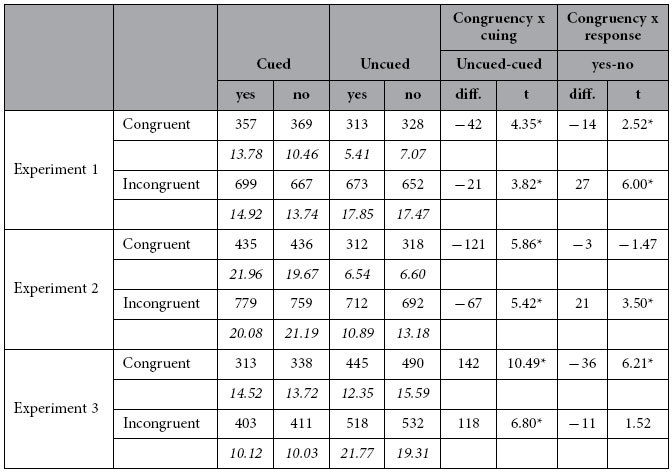
Structure-activity relationship of Btk inhibitors.

The two most rightward columns report the differences in ms between uncued/cued trials and yes/no responses, respectively. *t* values (df = 15) for the related comparisons are also reported; p values < .05 are identified by an asterisk (*).
