# Erratum: Disadvantageous associations: Reversible spatial cueing effects in a discrimination task

**DOI:** 10.1038/srep17242

**Published:** 2015-12-09

**Authors:** Daniele Nico, Elena Daprati

Scientific Reports 5: Article number: 1615610.1038/srep16156; published online: 11042015; updated: 12092015

In the HTML version of this Article, there is an error in Table. 1

“Structure-activity relationship of Btk inhibitors.”

should read:

“Mean Reaction Times (ms) and SE (in italics) for cued and uncued trials as a function of response (yes/no) and congruency between first shape and target (congruent=same shape incongruent=different shape) in the three experiments.”

